# ASPPH leadership on advancing institutional excellence in academic public health

**DOI:** 10.3389/fpubh.2026.1746598

**Published:** 2026-03-27

**Authors:** Laura Magaña, Dorothy Biberman, Miranda Bosse

**Affiliations:** Association of Schools and Programs of Public Health, Washington, DC, United States

**Keywords:** academic public health, framing the future, IDEA Institute, institutional excellence, public health education

## 1 Introduction

The Association of Schools and Programs of Public Health (ASPPH) is the voice of academic public health, representing over 150 graduate schools and programs of public health accredited and in the process of becoming accredited by the Council on Education for Public Health (CEPH) in seven countries. ASPPH also hosts an Undergraduate Network which represents over 90 undergraduate public health institutions. ASPPH is a leader in advancing institutional excellence by fostering systemic change in public health education, research, and practice, as evidenced by its innovative initiatives. ASPPH actively promotes safe and engaging environments for all students, faculty, and communities. The organization provides strategic guidance, tools, and resources to support schools of public health and public health programs in integrating institutional excellence policies into their curricula and institutional frameworks. ASPPH is committed to ensuring that future public health professionals are equipped to address and advance the health and wellbeing of all communities worldwide. This report serves as a retrospective, highlighting the past decade of ASPPH's work and progress in this space. It concludes by acknowledging the current landscape and the emerging challenges and opportunities that may shape the future of the field of academic public health.

## 2 Overview of institutional excellence initiatives

ASPPH provides evidence-based resources on public health education that support our members in training the current and future public health workforce. In 2015, ASPPH published the first Framing the Future reports to better prepare graduates for success in a changing world and global marketplace. These reports emphasized that public health graduates should receive training across a range of areas, including systems thinking, communication, health determinants, and the reduction and elimination of health outcomes disparities (1). In 2019, ASPPH released a Statement of Commitment to Zero Tolerance of Harassment and Discrimination, which declares that schools and programs of public health are opposed to all acts of harassment and discrimination (2). In developing this statement, ASPPH constituent groups emphasized that academic public health leaders must be at the forefront of creating institutions free of harassment and discrimination (3). These initiatives served as a foundation for subsequent actions.

In 2020, ASPPH released its Strategic Plan 2030, which sets out a bold and inclusive vision for academic public health. The Strategic Plan 2030 lists 13 goals, including advancing evidence-based solutions to public health challenges globally; advancing the journey on health equity in academic public health; advancing teaching, learning, research, practice, and scholarship; and enhancing ASPPH members' ability to graduate a diverse and inclusive student body (4). The same year, ASPPH established a task force on dismantling the conditions that enable and perpetuate discrimination and racism in academic public health (5). This led directly to the publication of “Dismantling Racism and Structural Racism in Academic Public Health: A Framework,” in 2021, which provides recommendations for ASPPH members (6).

In subsequent years, ASPPH developed two major frameworks to address critical public health issues, examined from the perspectives of education and training, research, policy and advocacy, and practice. Both frameworks emphasize “health for all” principles: “Responding to the Climate Change and Health Crisis: A Framework for Academic Public Health,” addressing health disparities as a cross-cutting theme (7), and “Gun Violence Prevention: An Academic Public Health Framework,” discussing how gun violence disproportionately affects certain communities (8). During this time, ASPPH also worked with the Robert Wood Johnson Foundation on the *Transforming Academia for Equity* (TAE) project, which serves as a collaborative initiative currently serving seven ASPPH member schools and programs of public health. TAE enables schools to transform academic spaces into safe, enriching, and engaging environments for the benefit of all faculty, staff, and students (9).

A significant milestone achieved in 2024 was the publication of the Framing the Future 2030 Reports. The series included three major reports: (1) “Building Inclusive Excellence through an Anti-racism Lens,” (2) “Transformative Approaches to Teaching and Learning,” and (3) “Fostering Community Partnerships for a Healthier World” (10). Also in 2024, ASPPH created the ASPPH Innovation, Discovery, and Excellence in Academia (IDEA) Institute. The ASPPH IDEA Institute will showcase and enhance our members' expertise in education, research, and practice, and will highlight data-driven decision-making in the field of institutional excellence in academic public health, working collectively to transform academic institutions that model institutional excellence (11).

## 3 Framing the future

ASPPH established the *original* Framing the Future Task Force in 2011 to better prepare graduates for success in a changing world and global marketplace. This comprehensive initiative to advance public health education was timed to conclude in 2015, with the 100-year anniversary of the seminal Welch-Rose report of 1915. The initiative produced seven seminal reports on employers, the MPH, the DrPH, undergraduate education, population health across all professions, community colleges, and the governmental public health workforce. Overall, the 2011–2015 Framing the Future called for transformation in public health degree programs, increased understanding of public health among clinical and other professions, and enhanced collaboration with governmental public health agencies (10).

In 2024, ASPPH released “Framing the Future: Education for Public Health 2030” (FTF 2030), a visionary project designed to prepare future public health professionals for a world of complex health challenges. There is an immense demand for public health education that is both adaptable and forward-thinking (12). Through this initiative, ASPPH embarked on a mission to enhance academic programs, ensuring they are well-equipped to foster the next generation of leaders with the objective of advocating for health and wellbeing for all communities globally. Building on the foundation of the original Framing the Future (10), FTF 2030 was rooted in the concept of representation from its inception, ensuring diverse voices participated in the expert panels so that the recommendations would be relevant across ASPPH institutions. Quality and transformative education for all is at the heart of FTF 2030.

This initiative comprised three expert panels, the first of which focused on promoting inclusive excellence. This report advances the recommendations of the previous framework. It makes five key recommendations for schools and programs of public health to advance health and wellbeing for everyone, everywhere, including creating inclusive teaching, learning, and working environments; ensuring accountability; increasing commitment among institutions; developing system-wide initiatives; and sharing initiatives across academic public health (13). The second report is focused on transformative approaches to teaching and learning. It proposes four recommendations for what and how schools and programs of public health teach, including centering civic engagement, collaboration, and community partnerships; grounding education in collective action to address the social determinants of health; using active learning to prepare diverse, practice-ready professionals; and assuring ongoing training in evidence-based frameworks (14). The third report is on fostering community partnerships, and the three recommendations are to position academic public health in partnership with communities, deploy strategies to support and sustain successful partnerships, and develop curricula to prepare learners with knowledge, skills, and mindsets for more effective partnering (15).

## 4 IDEA Institute

In June 2025, ASPPH launched the Institute for Innovation, Discovery, and Excellence in Academia (IDEA). The IDEA Institute serves as an interactive virtual resource portal and living classroom for academics, practitioners, and community members alike. The IDEA Institute fosters a synergistic exchange of learning in real time and offers a series of activities that span those of a collaborative digital library, an online classroom, and an exploratory laboratory designed for sharing innovations across public health institutions. Comprised of four guiding pillars, the Institute leverages the work happening throughout member schools and programs by showcasing initiatives in (1) Academic Public Health for the 21st Century, (2) Fostering Student Belonging and Engagement, (3) Teaching, Research, and Practice in Action, and (4) Interprofessional Community Engaged Scholarship (16).

In addition to the Institute, ASPPH developed and launched a people-centered chatbot, developed in partnership with Amazon Web Services (AWS), to support academic public health learners and leaders at all levels. ARIE, the Institute's AI Resource for Institutional Excellence, answers public health questions using curated, evidence-based content (17). ARIE was intentionally designed with the ethical and responsible use of Artificial Intelligence in mind, adhering to the IDEA Institute's core value of institutional excellence.

## 5 Discussion

The efforts of ASPPH over the past decade have centered on excellence, transformation, and access to education for all at the core of institutional practice. By embedding these principles into strategic planning, curricula, research, and community engagement, ASPPH has not only acknowledged the entrenched systems that exist within academia but also provided a clear roadmap to advance institutional excellence in academic public health.

From the 2015 Framing the Future reports to the 2024 launch of the IDEA Institute, ASPPH has systematically built a foundation for institutional excellence. The reports and frameworks were the outcomes of years of dialogue and discussion across academic public health institutions, practice partners, and communities, offering both philosophical grounding and practical guidance. The resulting frameworks continue to shape discussions in academic settings and influence the CEPH accreditation criteria. These efforts reflect a growing consensus that advancing institutional excellence for all depends on transforming the systems that prepare the public health workforce.

As the field confronts increasingly complex global health challenges, public health education must continue evolving. ASPPH's leadership underscores the urgent need to cultivate stimulating and dynamic learning environments where all students, faculty, and staff can thrive, and where innovation is driven with all populations in mind. Looking forward, the sustainability of these efforts depends on continuous institutional accountability, cross-sector collaboration, and the willingness to interrogate and change long-standing norms.

ASPPH's agenda, centered on institutional excellence, positions academic public health to actively shape a future in which all populations have a fair and just opportunity to attain their highest level of health and wellbeing. Through persistent and collective action, academic public health institutions can lead the charge toward a healthier and prosperous world.

As we reflect on a decade of progress, we must also acknowledge the challenges posed by today's political and social environment. These conditions have introduced uncertainty and, at times, imposed direct constraints on efforts to advance academic public health. Yet, despite these headwinds, the core mission and values of ASPPH remain unchanged. Our commitment to strengthening academic public health and preparing the next generation of public health leaders continues to guide our work.

In this evolving landscape, ASPPH remains engaged in sustained dialogue with schools and programs of public health to navigate the path forward—collectively. Meeting this moment requires both critical reflection and strategic adaptability. Accordingly, we are deliberately reassessing and refining our approaches to ensure that our efforts remain rigorous, relevant, and impactful.

The launch of the IDEA Institute marks an important step in this next phase, providing a platform to examine emerging challenges and opportunities, elevate evidence and promising practices, and amplify the contributions of public health scholars and communities. At a time when health challenges are increasingly complex and interconnected, the role of academic public health has never been more essential. The responsibility before us is unmistakable. We must continue to advance science, education, and practice—while boldly innovating and transforming academic public health—in steadfast service of the public's health. The decisions we make today will shape not only the strength of our institutions but also the health and wellbeing of communities for generations to come. Now is the moment to act, with clarity, courage, and collective resolve—because the future of public health depends on it.
